# Impact of the COVID-19 Pandemic on Surgical Colorectal Cancer Care in the Netherlands: a Multicenter Retrospective Cohort Study

**DOI:** 10.1007/s11605-021-04936-z

**Published:** 2021-02-23

**Authors:** Mando Filipe, Ellen de Bock, Ritch Geitenbeek, Djamila Boerma, Apollo Pronk, Joost Heikens, Milan Richir

**Affiliations:** 1 Department of Surgery, Cancer Centre, University Medical Centre Utrecht, Utrecht, The Netherlands; 2 Department of Surgery, Meander Medical Centre, Amersfoort, The Netherlands; 3 Department of Surgery, St. Antonius Hospital, Nieuwegein, The Netherlands; 4 Department of Surgery, Diakonessenhuis, Utrecht, The Netherlands; 5 Department of Surgery, Rivierenland Hospital, Tiel, The Netherlands

**Keywords:** COVID-19, Surgery, Pandemic, Complications, Colorectal cancer, Multicenter

## Introduction

Coronavirus disease 2019 (COVID-19) poses a challenge to routine healthcare practice.

The suspension of the national screening program combined with fewer referrals through primary care (the general practitioner) has led to fewer colorectal cancer diagnoses in the Netherlands.^1^ However, the consequences of the COVID-19 pandemic for colorectal cancer surgical care are unknown. Therefore, the aim of this study is to determine the impact of the COVID-19 pandemic on the surgical management of colorectal cancer.

## Methods

This retrospective cohort study included all patients with colorectal cancer who were operated on between March 9 and June 30, 2020, in four hospitals across the Netherlands. One of these was an academic hospital, and the other three were district general hospitals. The primary outcome was the number of colorectal cancer resections performed. Secondary endpoints were the number and risk factors of postoperative complications in patients undergoing colorectal cancer surgery.

Multivariate logistic regression analysis was performed to ascertain the risk of developing a complication (infections, bleeding, ICU admission, and/or anastomotic leakage) in patients who underwent colorectal cancer surgery after correcting for possible confounders. Odds ratio (OR) with 95% confidence intervals (CI) were used to quantify the risk of postoperative complications. Two-sided P values below 0.05 were considered statistically significant.

## Results

**Fig. 1:**
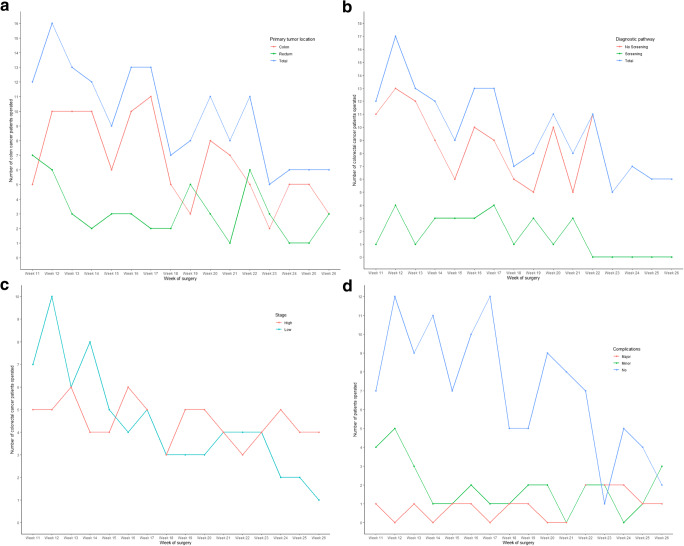
**a**–**d**. **a** Top left = number of colorectal cancer surgical procedures stratified by colon and rectum. **b** Top right = number of patients with colorectal cancer who had undergone surgical procedures from March 9 to June 30, 2020, with referral from national screening program or general practitioner. **c** Bottom right = number of colorectal cancer surgical procedures presented by tumor stage. Low = stage Tis, stage I and stage II. High = stage III and stage IV. **d** Bottom left = number of colorectal cancer surgical procedures presented by type of complications

One hundred sixty-two patients underwent colorectal resectional surgery for malignant pathology. There were 88 lower staged (stages 0–II) and 74 higher staged (stages III–IV) colorectal cancers. Of the 162 patients, 21 had been tested for COVID-19 (13.0%), two of whom tested positive (9.5%). Postoperative complications occurred in 46 (28.4%) patients. An overall reduction in the overall numbers of colorectal resections occurred over the time of the study (Fig. 1a). After week 22, no colorectal cancer resections were performed for patients diagnosed through the national screening program (Fig. 1b). Furthermore, an overall decrease was observed in the number of procedures performed for patients with colorectal cancer who were referred through primary care. The number of colorectal cancer procedures performed for patients diagnosed with low-stage tumors gradually declined during the course of the study period (Fig. 1c), while the high-stage tumors remained stable. During the study period, the number and risk (OR 1.03; 95% CI 0.94–1.12; P=0.573) of postoperative complications remained stable (Fig. 1d and Table 1).

**Table 1:** Multivariate analysis for the risk of complications

	Estimate	OR (CI)	Standard error	*Z* value	*P* value
Age in years	0.017	1.02 (0.98–1.05)	0.019	0.917	0.359
Sex					
Sex, female	NA	1.00 (reference)	NA	NA	NA
Sex, male	0.858	2.36 (0.93–5.98)	0.474	1.809	0.070
BMI	0.000	1.00 (0.91–1.10)	0.048	−0.01	0.992
Number of comorbidities	−0.086	0.92 (0.55–1.53)	0.261	−0.328	0.743
ASA	0.834	2.30 (1.00–5.29)	0.424	1.966	0.049
Setting					
Acute	NA	1.00 (reference)	NA	NA	NA
Elective	−1.38	0.25 (0.05–1.27)	0.825	−1.672	0.095
Screening	−2.382	0.09 (0.01–0.76)	1.073	−2.22	0.026
Location of primary tumor					
Colon	NA	1.00 (reference)	NA	NA	NA
Rectum	0.693	2.00 (0.72–5.53)	0.519	1.335	0.182
Neo-adjuvant therapy	0.415	1.51 (0.41–5.62)	0.669	0.62	0.536
Tumor stage	0.008	1.01 (0.66–1.55)	0.218	0.038	0.970
Week of surgery	0.025	1.03 (0.94–1.12)	0.044	0.563	0.573

Table showing results of the multivariate analysis for the risk of patients developing postoperative complications. Abbreviations: *ASA* American Society of Anesthesiologist, *BMI* body mass index, *CI* confidence interval, *COVID-19* Coronavirus disease 2019, *NA* not applicable, *OR* odds ratio

## Discussion

The current study showed a decline of colorectal surgical procedures during the study period. This appears to be a result of the suspension of the national colorectal screening program as well as a reduction in the number of referrals from primary care. The result of this has been a decrease in the apparent incidence of lower staged tumors. This is unsurprising, since colorectal cancers diagnosed at the national screening program are detected at an early stage compared to patients who present with colorectal-related symptoms.^1^,^2^ The national screening program restarted on June 1, 2020. However, this has not (yet) led to an increase in colorectal surgical procedures performed. Possible reasons could be that the screening program has not yet fully started (70% of the invitation rate). Additionally, the colorectal cancer screening program consists of a fecal immunochemical test (FIT) in the first instance. Only if FIT is positive will an endoscopy be offered.^3^

This study also showed that the number of patients presenting with symptomatic colorectal cancer (non-screened patients) decreased during the COVID-19 period which could be a representation of the decreased referrals by primary care.

Since the ongoing COVID-19 pandemic puts an enormous strain on healthcare systems worldwide, this study showed that, if necessary, suspension of the national screening program is acceptable as long as explicit attention is paid to the continuation primary care referrals.
